# Relationship Involving Sexual Function, Distress Symptoms of Pelvic Floor Dysfunction, and Female Genital Self-Image

**DOI:** 10.1055/s-0043-1772474

**Published:** 2023-10-16

**Authors:** Guilherme Tavares de Arruda, Gabrielle Peres Paines, Bianca Rangel da Silva, Lauren Xavier Pairé, Hedioneia Maria Foletto Pivetta, Melissa Medeiros Braz, Janeisa Franck Virtuoso

**Affiliations:** 1 Departamento de Fisioterapia, Universidade Federal de São Carlos, São Carlos, SP, Brazil; 2 Departamento de Fisioterapia e Reabilitação, Universidade Federal de Santa Maria, Santa Maria, RS, Brazil; 3 Departamento de Fisioterapia, Universidade Federal de Santa Catarina, Araranguá, SC, Brazil

**Keywords:** women, sexual function, pelvic floor dysfunction, genital self-image, mulheres, função sexual, disfunção do assoalho pélvico, autoimagem genital

## Abstract

**Objective** To assess the relationship involving sexual function (SF), the distress symptoms caused by pelvic floor dysfunction (PFD), and female genital self-image (GSI).

**Materials and Methods** We assessed the GSI, SF and PFD distress symptoms by the Female Genital Self-Image Scale (FGSIS), the Female Sexual Function Index (FSFI), and the Pelvic Floor Distress Inventory (PFDI-20) respectively. Data were analyzed by multiple linear regression.

**Results** Among the 216 women (age: 50.92 ± 16.31 years) who participated in the study, 114 were sexually active in the previous 4 weeks. In the total sample ( *p* < 0.001; adjusted R ^2^ = 0.097) and among sexually active women ( *p* = 0.010; adjusted R ^2^ = 0.162), the distress symptoms caused by pelvic organ prolapse (POP) were related to the GSI. Among sexually active women, sexual desire also was related to the GSI ( *p* < 0.001; adjusted R ^2^ = 0.126).

**Conclusion** The findings of the present study provide additional knowledge about female GSI and suggest that SF and POP distress symptoms should be investigated together with the GSI in the clinical practice.

## Resumo

**Objetivo** Avaliar a relação entre a função sexual (FS), o incômodo provocado pelos sintomas de disfunção do assoalho pélvico (DAP) e a autoimagem genital (AIG) feminina.

**Materiais e Métodos** A AIG, a FS e incômodo causado pelos sintomas de DAP foram avaliados pela Genital Self-Image Scale (FGSIS), pelo Female Sexual Function Index (FSFI) e pelo Pelvic Floor Distress Inventory (PFDI-20), respectivamente. Os dados foram analisados por regressão linear múltipla.

**Resultados** Das 216 mulheres (idade: 50,92 ± 16,31 anos) que participaram do estudo, 114 eram sexualmente ativas nas últimas 4 semanas. Na amostra total ( *p* < 0,001; R ^2^ ajustado = 0,097) e entre as mulheres sexualmente ativas ( *p* = 0,010; R ^2^ ajustado = 0,162), o incômodo provocado pelos sintomas de prolapso de órgãos pélvicos (POP) relacionou-se à AIG. Entre as mulheres sexualmente ativas, o desejo sexual também se relacionou à AIG ( *p* < 0,001; R ^2^ ajustado = 0,126).

**Conclusão** Os achados deste estudo fornecem conhecimento adicional sobre a AIG feminina e sugerem que a FS e o incômodo causado pelos sintomas de POP devem ser investigados juntamente com a AIG na prática clínica.

## Introduction

Genital self-image (GSI) is defined as the feelings and opinions of an individual about their own genitals, 1 and it is an important component of body image. 2 In women who are dissatisfied with their genitals, the level of anxiety increases when exposing them during sexual activity, 1 which can decrease the sensation of pleasure and generate pain during penetration. 3 4 Therefore, studies indicate the relationship between GSI and sexual function (SF), including its six domains: sexual desire, arousal, lubrication, orgasm, sexual satisfaction, and pain. 1 2 3 4 5 Thus, a worse GSI can interfere with quality of life, generating cases of sexual dysfunction and reducing the frequency of gynecological exams. 4 6

In women with pelvic floor dysfunction (PFD), including urinary incontinence (UI), pelvic organ prolapse (POP), and anorectal disorders, the SF and GSI can be negatively influenced. This can lead to cases of depression, decreased sexual activity, social isolation, and decreased quality of life. 3 7 8 9 It seems that distress symptoms, including those caused by PFD, as well as anxiety, and depression, negatively affect female GSI. 8 Among women with POP, there is a relationship involving the SF, the GSI and the severity of the POP, as the concern about showing the prolapsed genitalia to the sexual partner during sexual activity involving penetration and oral sex generates fear and insecurity in the woman, which contributes to a worse SF, especially in terms of sexual desire and satisfaction. 7

Although studies on the relationship involving the SF, female GSI and PFD is scarce in the literature, 7 8 understanding the impact of the SF and the distress symptoms caused by PFD on female GSI is necessary, because the PFD distress symptoms affect the quality of sex life. 9 Once health professionals have a better understanding of this relationship, they can promote care and treatment strategies aimed at this population. Thus, the aim of the present study was to assess the relationship involving the SF, the PFD distress symptoms, s and female GSI. Therefore, we have hypothesized that the best SF would be related to the best GSI, 2 and that the greater the PFD distress symptoms, the worse the GSI. 7

## Materials and Methods

The present is a cross-sectional and observational study approved by the institutional Ethics Committee (CAAE: 13189919,0,0000,0121; n° 3,437,754) and carried out with a sample of women from three cities in the states of Rio Grande do Sul and Santa Catarina, Southern Brazil. Women were intentionally invited to participate in the study between November 2019 and March 2020. Data collection was interrupted due to the coronavirus disease 2019 (COVID-19) pandemic. We invited women to participate while they waited for a medical appointment at the General Medicine Ward, which were selected due to convenience and because it receives the largest number of people. Trained researchers interviewed the participants face-to-face in a private room. The present study followed the Strengthening the Reporting of Observational Studies in Epidemiology (STROBE) checklist. 10

Women aged over 18 years who could read and write in Brazilian Portuguese were included. We excluded women who reported symptoms of urinary tract infection in the previous week (pain and burning sensation when urinating), pregnant women, those with ≤ 6 months of puerperium, and wheelchair users.

To characterize the sample, a questionnaire developed by the authors was applied, and it included questions about age, level of schooling, skin color, smoking, alcohol intake, physical activity, number of pregnancies, vaginal deliveries, cesarean sections, abortions, gynecological surgery, and episiotomy. A digital scale and a stadiometer were used to measure weight and height respectively, to calculate the body mass index (BMI).

To evaluate female GSI, we used the Female Genital Self-Image Scale (FGSIS) validated for Brazilian Portuguese, which showed excellent values in terms of internal consistency (α = 0.81), intra- (intraclass correlation coefficient [ICC] = 0.89) and interobserver (ICC = 0.83) reliability in its validation in Brazil. The FGSIS contains 7 items whose scores range from 1 (strongly disagree) to 4 (strongly agree). The total FGSIS score ranges from 7 to 28 points, and the higher the score, the better the GSI. 12

Sexual function was assessed by the Female Sexual Function Index (FSFI) regarding the previous 4 weeks through 19 items divided into 6 SF domains: sexual desire, arousal, lubrication, orgasm, sexual satisfaction, and pain. This instrument was validated among Brazilian women, with excellent values for internal consistency for the total score (α = 0.96) and for test-retest reliability (ICC = 1.00). The total FSFI score ranges from 2 to 36 points, and it is the sum of the scores On each domain multiplied by a factor that equalizes the influence of each weighted score on the total score; the higher the score, the better the SF. With the exception of the sexual desire domain, all other domains can only be assessed in women who have been sexually active in the previous four weeks. 13

To assess the PFD distress symptoms, we used the Pelvic Floor Distress Inventory (PFDI-20) which was validated among Brazilian women with adequate values for internal consistency (α ≥ 0.70) and test-retest reliability (ICC ≥ 0.70). This instrument contains twenty items divided into three subscales to assess the POP (through the Pelvic Organ Prolapse Distress Inventory, POPDI-6), anorectal (through the Colorectal-Anal Distress Inventory – CRADI-8) and urinary (Urinary Distress Inventory – UDI-6) distress symptoms. The calculation of the score on each subscale is made by the average of the 6 or 8 items multiplied by 25, and the higher the score, the worse the distress symptoms. The total PFDI-20 score is the sum of the scores on each subscale. 14

Initially, the data were considered to have a non-parametric distribution by the Kolmogorov-Smirnov test. Thus, we used the Spearman correlation coefficient (rho) to assess the correlation regarding the GSI, the SF domains, and the PFD distress symptoms. The strength of the correlation was determined by the Cohen criteria: 15 r ≤ 0.29–weak correlation; r ranging from 0.30 to 0.49–moderate correlation; and r ≥ 0.50–a strong correlation. The coefficient of determination (R ^2^ ) was used to measure the effect of the correlation; it is presented as a percentage, and it expresses the proportion of variation in one measure that is explained by the variation in another measure. Multiple linear regression with the orward insertion method was used to determine the variable that best predicts the GSI. We presented the regression with the F values (degrees of freedom), *p* -value and adjusted R ^2^ . Variables with *p* < 0.05 in the correlation analysis entered the regression model. In all tests, we adopted *p* < 0.05. All analyzes were performed using the IBM SPSS Statistics for Windows (IBM Corp., Armonk, NY, United States) software, version 22.0.

The calculation of the sample power was performed after we obtained the results based on the R ^2^ of the correlation between the GSI and the POP distress symptoms in the total sample. For this calculation, we used R ^2^ = 0.097, α = 0.05, and n = 216 in a multiple linear regression model in G*Power 3.1.9.7. Thus, the power of the sample was of 99%.

## Results

In total, 262 women participated in the study; however, 35 were excluded due to reports of symptoms or a diagnosis of lower urinary tract infection in the previous week, 10, for not completing the interview, and 1, for being pregnant. Thus, data from 216 women were analyzed. Of these, 114 (52.78%; age: 43.91 ± 14.60 years) were sexually active in the previous 4 weeks. Table 1 shows the characteristics of the total sample (age: 50.92 ± 16.31 years) and of the sexually active subgroup (age: 43.91 ± 14.60 years); their respective GSI values were of 22.37 ± 4.25 and 22.95 ± 3.81 points. Most women in the total sample and in the sexually active subgroup were white (68.98% and 69.30% respectively), non-smokers (90.74% and 89.47% respectively), had not had an episiotomy (55.09% and 59.65% respectively), and did not practice physical activity (58.33% and 56.14% respectively). As for the distress symptoms among the total sample and the sexually active subgroup respectively, the mean values are as follows: POP –10.82 ± 17.17 and 9.10 ± 12.84 points; anorectal –16.07 ± 19.00 and 13.92 ± 16.09 points; urinary – 19.79 ± 25.16 and 16.04 ± 21.08 points; and PFD – 46.69 ± 52.44 and 39.07 ± 39.21 points ( Table 1 ).

**Table 1:** Characteristics of the study participants

Characteristics	Total sample (n = 216): mean ± SD or n (%)	Sexually active women (n = 114): mean ± SD or n (%)
Age (years)	50.92 ± 16.31	43.91 ± 14.60
Schooling (years)	10.86 ± 5.83	12.02 ± 5.21
Skin color		
* White*	149 (68.98)	79 (69.30)
* Black*	17 (7.87)	12 (10.53)
* Brown*	43 (19.91)	19 (16.67)
* Other*	07 (3.24)	04 (3.50)
Body mass index (kg/m ^2^ )	27.49 ± 7.04	26.60 ± 6.75
Smoker		
* No*	196 (90.74)	102 (89.47)
* Yes*	20 (9.26)	12 (10.53)
Number of pregnancies	2.44 ± 1.84	1.89 ± 1.52
Number of vaginal deliveries	1.40 ± 1.69	0.97 ± 1.39
Number of cesarean sections	0.70 ± 1.00	0.68 ± 0.98
Number of abortions	0.35 ± 0.68	0.26 ± 0.58
Episiotomy		
* No*	119 (55.09)	68 (59.65)
* Yes*	95 (43.98)	46 (40.35)
*Do not remember*	02 (0.93)	0
Physical activity		
* No*	126 (58.33)	64 (56.14)
* Yes*	90 (41.67)	50 (43.86)
Genital self-image	22.37 ± 4.25	22.95 ± 3.81
Sexual desire	–	3.55 ± 1.23
Arousal	–	4.21 ± 1.28
Lubrication	–	4.97 ± 1.30
Orgasm	–	4.71 ± 1.40
Sexual satisfaction	–	5.11 ± 1.23
Pain	–	5.09 ± 1.30
Overall sexual function	–	27.65 ± 6.41
Distress symptoms		
*Pelvic organ prolapse*	10.82 ± 17.17	9.10 ± 12.84
*Anorectal*	16.07 ± 19.00	13.92 ± 16.09
*Urinary*	19.79 ± 25.16	16.04 ± 21.08
*Pelvic floor dysfunction*	46.69 ± 52.44	39.07 ± 39.21

Abbreviation: SD, standard deviation.

Table 2 shows the correlations regarding the GSI, the SF and the PFD distress symptoms for the total sample and for the subgroup of sexually active women. Among the sexually active women, except for the lubrication domain, all other domains and the overall SF were significantly correlated with GSI. These correlations were moderate for sexual desire (rho = 0.338), arousal (rho = 0.374), orgasm (rho = 0.303), and overall SF (rho = 0.377), and weak for sexual satisfaction (rho = 0.264) and pain (rho = 0.275). In both samples, the worse the POP (rho = -0.284), urinary (rho = -0.287) and PFD (rho = -0.293) distress symptoms, the worse the GSI, and these correlations are weak. The anorectal distress symptoms had a significant and negative correlation only in the total sample (rho = -0.202). The variable that best explained the variation in GSI in the total sample was the PFD distress symptoms (8.6%). Among the sexually active women, overall SF was the variable that best explained the variation in GSI (14.2%).

**Table 2:** Correlation regarding genital self-image, sexual function, and the distress symptoms of pelvic floor dysfunction

	Genital self-image			
	Total sample (n = 216)		Sexually active women (n = 114)	
	rho	R ^2^	rho	R ^2^
Sexual desire	–	–	0.338**	0.114
Arousal	–	–	0.374**	0.140
Lubrication	–	–	0.089	0.008
Orgasm	–	–	0.303*	0.092
Sexual satisfaction	–	–	0.264*	0.070
Pain	–	–	0.275*	0.076
Overall sexual function	–	–	0.377**	0.142
Distress symptoms				
*Pelvic organ prolapse*	-0.284**	0.081	-0.239*	0.057
*Anorectal*	-0.202*	0.041	-0.131	0.017
*Urinary*	-0.287**	0.082	-0.232*	0.054
*Pelvic floor dysfunction*	-0.293**	0.086	-0.241*	0.058

Notes: R ^2^ , coefficient of determination; rho, spearman correlation coefficient; * *p* < 0.05; ** *p* < 0.001.

The POP distress symptoms significantly influenced the GSI in the total sample (F[1, 214] = 23.898; *p* < 0.001; adjusted R ^2^ = 0.097). According to table 3 , in the total sample, for each increase of 1 point in the GSI, there was a decrease of 0.079 points in the POP distress symptoms, a variable that explained 9.7% of the GSI variation. For sexually active women, a significant influence of the POP distress symptoms on the GSI was also observed (F[2, 111] = 11.918; *p* = 0.010; adjusted R ^2^ = 0.162). For every one-point increase in the GSI, 1.059 points were increased in sexual desire and 0.067 points were decreased in the POP distress symptoms distress. The variable that most explained the variation in GSI (16.2%) was the POP distress symptoms. The other domains of SF and the PFD distress symptoms did not show a significant relationship ( *p* > 0.05) with the GSI in both groups.

**Table 3:** Multiple linear regression of the predictors of genital self-image of

		Total sample (n = 216)				
Variables	Non-standardized beta	Standardized beta	t	*p*	R ^2^	ΔR ^2^
Distress symptoms						
*Pelvic organ prolapse*	-0.079	-0.317	-4.898	< 0.001	0.097	0.101
*Anorectal*	-0.001	–	-0.012	0.991	0.001	–
*Urinary*	-0.109	–	-1.318	0.189	0.090	–
*Pelvic floor dysfunction*	-0.125	–	-0.976	0.330	0.67	–
		**Sexually active women (n = 114)**				
**Variables**	**Non-standardized beta**	**Standardized beta**	**t**	**p**	** R ^2^**	** ΔR ^2^**
Sexual desire	1.059	0.343	3.976	< 0.001	0.126	–
Arousal	0.198	–	1.545	0.125	0.145	–
Orgasm	0.138	–	1.257	0.211	0.118	–
Sexual satisfaction	0.148	–	1.322	0.189	0.125	–
Pain	0.143	–	1.492	0.138	0.140	–
Overall sexual function	0.182	–	1.270	0.207	0.120	–
Distress symptoms						
*Pelvic organ prolapse*	-0.067	-0.226	-2.617	0.010	0.162	0.051
*Urinary*	-0.099	–	-1.098	0.275	-0.104	–
*Pelvic floor dysfunction*	-0.162	–	-1.818	0.072	-0.170	–

Notes: R ^2^ , coefficient of determination; ∆R ^2^ , variation of the coefficient of determination; t, t-test.

## Discussion

The study investigated the relationship involving SF, PFD distress symptoms, and GSI. In the multivariate analysis, our findings showed that the POP distress symptoms influenced the GSI of the total sample and of the subgroup of sexually active women, and that sexual desire influenced the GSI among sexually active women. In the bivariate analysis, better scores on the domains of sexual desire, arousal, orgasm, sexual satisfaction, and overall SF were related to better GSI among sexually active women. On the other hand, better GSI was related to worse urinary, POP and PFD distress symptoms in this subgroup of women. In the total sample, better GSI was related to worse urinary, anorectal, POP, and PFD distress symptoms.

The relationship between POP and female GSI has been discussed previously, especially given the severity of the POP. 7 The more severe the POP, the worse the GSI, as the image of an organ coming out of the vagina can be strange for the woman and the sexual partner. This can also interfere with their intimate relationship on the female SF in all domains. 7 8 16 In addition, maintaining a satisfying sex life contributes to physical and mental well-being, which is essential for a positive GSI. Although the GSI can directly influence intimacy, attention, and trust during sexual intercourse, 17 women with negative GSI can maintain an active sex life to avoid personal insecurities, negative emotions, and partner conflicts. 18

Although in the present study we did not assess POP severity, its distress symptoms were assessed through a subjective questionnaire, and they influenced the GSI among the subgroup of sexually active women. It is possible that feelings of shame and concern about the genital image are also related to the women's concern that the POP may worsen during sexual activity. 7 Also, in the current study the better the scores on the domains of desire, arousal, orgasm, sexual satisfaction, pain, and overall SF, the better the GSI among sexually active women. Similar results were found in a study 7 conducted in Indonesia, in which a low GSI was related to cases of sexual dysfunction in women with POP. In a study 8 with Israeli women with PFD, the GSI predicted total SF and sexual desire. In the same study, 8 when the SF increased, the GSI also increased.

While female GSI encompasses a woman's feelings about her genitals, the genitalia reflects a person's sexual experience, and it can influence SF. Due to the complexity of the PFD and its negative relationship with GSI, the SF also suffers a negative impact. The aforementioned Israeli study 8 showed that infrequent orgasm, decreased arousal, and increased dyspareunia are present in women with all types of PFD. 8

The present study is on issues scarcely addressed in the literature regarding female GSI. We separately examined GSI behavior in the subset of sexually active women in different analysis. However, there were some limitations. First, we assessed PFD distress symptoms subjectively by the PFDI-20, which also made it impossible to assess POP severity for the analysis of the relationship with GSI and the inclusion of women diagnosed with PFD. Second, the convenience sample of women waiting for a medical appointment makes it difficult to generalize the results of the present study. Third, we did not investigate the diagnosis of depression or the use of antidepressants, as they may influence women's perception of their body/genital image. 1 Finally, the cross-sectional design does not enable the determination of the cause and effect of the variables investigated. Thus, future studies with longitudinal design should consider the objective assessment of PFD distress symptoms to assess the causes and effects regarding GSI, SF and PFD distress symptoms.

## Conclusion

In the present study, female GSI was negatively influenced by the POP distress symptoms and positively influenced by sexual desire. These findings provide additional knowledge about female GSI and suggest that SF and POP distress symptoms should be investigated alongside GSI in the clinical practice. Thus, it will be possible to collect precious information about female GSI and plan the appropriate treatment aimed at sexual dysfunction, since SF is a component of quality of life.
